# Methods of Synthesis, Properties and Biomedical Applications of CuO Nanoparticles

**DOI:** 10.3390/ph9040075

**Published:** 2016-11-30

**Authors:** Madalina Elena Grigore, Elena Ramona Biscu, Alina Maria Holban, Monica Cartelle Gestal, Alexandru Mihai Grumezescu

**Affiliations:** 1 Department of Biomaterials and Medical Devices, Faculty of Medical Engineering, University Politehnica of Bucharest, Bucharest 060042, Romania; grigore.madalina10@gmail.com (M.E.G.); biscu.ramona@hotmail.com (E.R.B.); 2 Division of Earth, Environmental and Life Sciences, Research Institute of the University of Bucharest (ICUB), Bucharest 060042, Romania; alina_m_h@yahoo.com (A.M.H.); grumezescu@yahoo.com (A.M.G.); 3 Department of Science and Engineering of Oxide Materials and Nanomaterials, Faculty of Applied Chemistry and Materials Science, University Politehnica of Bucharest, Bucharest 060042, Romania; 4 Department of Infectious Diseases, College of Veterinary Medicine, University of Georgia Athens, Athens, GA 30602, USA

**Keywords:** CuO, antimicrobial nanoparticles, synthesis, biomedical nanostructures

## Abstract

This study aims to provide an updated survey of the main synthesis methods of copper oxide (CuO) nanoparticles in order to obtain tailored nanosystems for various biomedical applications. The synthesis approach significantly impacts the properties of such nanoparticles and these properties in turn have a significant impact on their biomedical applications. Although not widely investigated as an efficient drug delivery system, CuO nanoparticles have great biological properties including effective antimicrobial action against a wide range of pathogens and also drug resistant bacteria. These properties have led to the development of various approaches with direct applications to the biomedical field, such as tailored surfaces with antimicrobial effect, wound dressings and modified textiles. It is also believed that these nanosystems could represent efficient alternatives in the development of smart systems utilized both for the detection of pathogens and for the treatment of infections.

## 1. Introduction

Research interest in nanomaterials has increased exponentially thanks to their unique chemical and physical features, different of those of their bulk materials, including but not limited to diffusivity, electrical resistivity, electrical conductivity, strength and hardness, chemical reactivity and diverse and versatile biological activity [1,2].

Interest has especially increased in the case of metal oxide nanoparticles, because these particles are widely used as industrial catalysts, chemical sensing devices, in medical applications, disinfection, as antimicrobials, fillers, opacifiers, catalysts, semiconductors and they are also useful in the development of cosmetics and microelectronics [1,2,3,4,5].

Metal oxide nanoparticles, such as copper oxide (CuO), have attracted attention mostly because of their antimicrobial and biocide properties and they may be used in many biomedical applications [6,7]. Copper oxide is a semiconductor metal with unique optical, electrical and magnetic properties and it has been used for various applications, such as the development of supercapacitors, near-infrared filters, in magnetic storage media, sensors, catalysis, semiconductors, etc. [8,9,10].

One of the most important parameters in the synthesis of these nanoparticles is the control of particle size, morphology and crystallinity and in order to achieve this goal, different synthesis methods were developed; some of the most investigated approaches include the sonochemical method, the sol-gel method, laser ablation, the electrochemical method, chemical precipitation and surfactant-based techniques [2,5,11,12,13].

Even though CuO nanoparticles (CuO NPs) have proved their use in biomedical applications; the major disadvantage for their use on the medical field is due to their potentially toxic effects [7,14,15,16]. CuO NPS may be toxic for mammalian cells as well as for vertebrates and invertebrates. The main toxicity process relies on the increased production of reactive oxygen species [17]. These nanoparticles thus induce oxidative stress in human pulmonary epithelial cells, promote toxicity and can damage DNA and mitochondria [7,15,18].

## 2. Methods of Synthesis for Biomedical CuO Nanoparticles

The synthesis approaches of CuO NPs have advanced significantly in the last ten years because of their important biomedical and industrial applications [19]. The synthesis technique is important for the properties of the final nanosystem, since it may control the size and morphology of the nanoparticles. Also, these nanoparticles present various optical and magnetic properties, mechanical strengths and electrical resistivity, which differ from the characteristics of bulk solid material. Several methods for the synthesis of CuO NPs have been used, and the most relevant approaches, along with the typical resulting particle sizes, are listed in Table 1.

### 2.1. Electrochemical Method

The electrochemical method was invented by Switzer as a way to synthesize ceramic films. Since then this method has been continuously used for the preparation of nano-metal oxides such as ZnO, CuO, etc. The first reported CuO nanocrystals were prepared by using Cu as a sacrificial anode [20,21].

The electrochemical method is based on reactions occurring between the electrode and the electrolyte. With this approach electrodeposition occurs on a small portion of the electrode, because chemical potentials are developed on its surface.

The electrochemical method is included in the group of soft chemical techniques that produce copper oxide nanoparticles [5]. One of the most notable advantages of this method is the ability to control the morphology and size of the resulting CuO NPs by modifying the temperature, time, current density, composition or voltage. Zhang et al. synthesized CuO nanospindles and nanorods by varying the density from 5 mA·cm^−2^ to 10 mA·cm^−2^ and then to 20 mA·cm^−2^. By changing the electrolytic solvent, these authors obtained CuO nanorods with diameters between 20 nm and 50 nm and with lengths of 200 nm to 300 nm [8]. Jadhav et al. also synthesized CuO NPS by applying the electrochemical method using a copper sheet as anode and a platinum sheet as a cathode [22].

Katwal et al. also described a CuO NPs manufacturing process using the electrochemical method under different reaction conditions. In this experiment the usual procedure was used, where the electrodes, copper plate and inert platinum were fixed at 1 cm. Also, the approach included a supporting electrolyte, which was added to acetonitrile and to a water to methanol solution in 12:1 molar ratio at room temperature. The dark brown precipitate could be centrifuged, washed, dried and finally the material was be easily calcined and characterized by different methods. This approach also revealed that physic and chemical properties of the nanostructures (including size) might be modified by controlling various parameters of the reaction and molar ratios of the utilized chemicals [5].

### 2.2. PEG–Dependent Synthesis

Polyethylene glycol (PEG) is a cheap non-ionic surfactant that is used for the synthesis of metal oxides [13]. It is also used in many biomedical applications, especially drug delivery, since it offers a good biocompatibility to the whole structure which contains it [9]. PEG 400 is the most commonly utilized variant due to its lower toxic [23]. For example, Ranjbar-Karimi et al. used this surfactant to study the effects induced by its presence on the dimension and morphology of CuO NPs. CuO NPs were prepared with the addition of sodium hydroxide solution at various concentrations and solutions of copper acetate in ethanol/water. The sample, which consisted of Cu(OAc)_2_·2H_2_O 50 mL (0.05 M) and NaOH 100 mL (0.1 M) that were sonicated for 1 h with 30 W ultrasound power. The method led to the production of nanoparticles with relatively homogenous dimensions (with an average diameter of 70 nm) [24].

Also, it was demonstrated by different studies that PEG has a significant effect on the size of CuO NPs. Vidyasagar et al. synthesized CuO NPs by mixing copper chloride, sodium hydroxide and PEG 400. The product was washed with ethyl alcohol to remove the PEG 400 and then it was dried. The resulting solid was calcined at 400 °C, 600 °C and 800 °C.

Samples calcined at 800 °C formed uniform particles, with sizes between 400 and 454 nm, while the samples calcined at 400 °C presented a nanoparticle size of approximately 65 nm. It was observed that the increase in temperature was proportional to the particle agglomeration [23]. In comparison, when Lashanizadegan and Erfaninia synthesized Ag/CuO nanoparticles and CuO nanorods by using PEG 400 and PEG 6000 it was observed that Ag/CuO nanoparticles synthesized with 10 mL PEG 400 or 20 mL PEG 400 have uniform morphology. However, the best morphology and distribution was obtained with PEG 6000 [25].

### 2.3. Sonochemical Method

The sonochemical method is a simple process that follows three steps: (1) formation, (2) development, (3) the implosive collapse of the obtained microcavities. The method involves the application of ultrasound during the synthesis of the product [8,26,27].

Suleiman et al. obtained CuO NPs of different morphology by using cupric acetate as a precursor and poly(vinylpyrrolidone) (PVP), acting as a reducing agent by applying a sonochemical method [28]. In comparison, Karunakaran et al. synthesized two sets of samples by applying the sonochemical method. The first set was formed from CuO NPs and cetyltrimethylammonium bromide (CTAB) and the second from CuO NPs without CTAB, after sonication and calcination. The results revealed that in the absence of CTAB, the nanoparticles have irregular shape and particle agglomeration was favored. Karunakaran et al. reported that the presence of CTAB promotes the crystal formation of CuO [29]. Also, Wongpisutpaisan et al. synthesized CuO NPs using the sonochemical method and then the product was calcined at various temperatures between 400 and 700 °C for 2 h. It was observed that at 400 and 500 °C the formation of CuO NPs was proved to be incomplete.

However, at 600 °C and 700 °C the authors observed the crystallization and the formation of uniform nanoparticles [30]. This method was used for the design of CuO NPs intended for medical applications. For example, Abramov et al. synthesized CuO NPs, which were used to coat medical cotton wound dressings and bandages by using the sonochemical method. It was reported that this combination was able to avoid microbial colonization and even kill various clinically relevant microorganisms such as *Escherichia coli*, which are reported to colonize the wounds of patients with skin lesions and require the usage of cotton dressings [31]. Also, Perelshtein et al. used the sonochemical method for the synthesis of CuO NPs to obtain coated textiles. These nanocoatings have shown an efficient antibacterial activity [32], therefore they are considered for the development of further biomedical applications, especially in the design of anti-infective surfaces, medical devices and anti-biofilm approaches.

### 2.4. Sol-Gel Method

The sol-gel technique is a simple and relatively fast method and therefore it is widely used in the design of nanoparticles [33]. This method is applied often as it ensures the rigorous control of the nanoparticle size. The method was optimized in order to obtain nanoparticles with dimensions ranging between 10 and 40 nm. Karthik et al. synthesized CuO NPs with dimensions of 25 nm by a sol-gel method [34]. The physical properties of CuO NPs also depend on the applied sol-gel method and the calcination time [28].

Moreover, in the case of sol-gel method, the size of nanoparticles is proportionally related with the temperature, physical conditions are very important for the design of functional nanoparticles using this approach [35]. Also by a sol-gel method, Jayaprakash et al. synthesized uncapped and capped CuO NPs by using ethylene diaminetetraacetic acid (EDTA). The capping agent was used to control the dimensions of CuONPs. Uncapped CuO NPs were synthesized with Cu(CH_3_COO)_2_·H_2_O and urea. It was reported that this method allows the fine control of morphology and shape of the nanoparticles [33].

### 2.5. Other Synthetic Methods

Other methods for the synthesis of CuO NPs have been developed; such as hydrothermal approach [31], thermal oxidation method [8], alcohol-thermal synthesis [36], liquid ammonia [37] and microwave-assisted synthesis [29].

Direct thermal decomposition method is broadly used for the synthesis of CuO NPs. One approach consists in adding sodium carbonate to copper sulfate and by calcination spherical CuO NPs are formed [28].

CuO NPs obtained by using the thermal plasma technique have been proved to display improved properties which may be helpful in their biomedical applications. For example, such nanostructures seem to have enhanced antimicrobial activity against drug resistant bacteria, while maintaining an acceptable biocompatibility and small dimensions [38].

**Figure 1:**
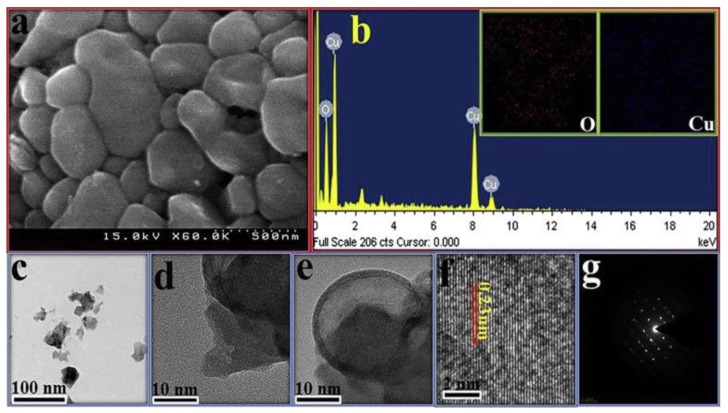
Microscopic images of CuO NPs synthesized by a green method: (**a**) scanning electron microscopy (SEM) image; (**b**) EDAX spectrometry of the CuO NPs; inset: elemental mapping of oxygen and copper; (**c**–**e**) transmission electron microscopy (TEM) images at different magnifications; (**f**) high magnification view of the CuO NPs; and (**g**) Selected Area Electron Diffraction (SAED) pattern of the CuO NPs [39].

In the last years, green syntheses of nanoparticles, including of CuO NPs with biomedical purpose were intensively studied. Green synthesis is a preferred alternative of synthesis since is safer for the biological systems, environmental friendly and physical and chemical characteristics of nanoparticles are still suitable for biomedical use (Figure 1) [39].

## 3. Properties

The properties of the CuO NPs depend on the synthesis method selected and they are very important for their applications in various areas, such as biomedical research, which is the most predominant. The most important feature is the size of the nanoparticles (which may be controlled during the synthesis) because it allows the tailored modeling of their optical, catalytic, electrical, and biological properties [5]. These properties make them useful for multiple applications such as the development of cosmetics, pharmacological alternatives, paints, coatings, etc. [17]. Therefore, the applied synthesis method, the modulation of the reaction parameters and the composition of bulk material represent key aspects in the direct control of size and direct or indirect control of other important physical, chemical and biological properties.

### 3.1. Optical Properties

The optical properties of CuO NPs are significantly influenced by the temperature, size and morphology [8,13,23].

For example, one useful technique is UV–Vis absorption spectroscopy, which is very important as it provides essential information about the optical properties of the material. El Sayed et al. studied the optical properties of thin films made of carboxymethyl cellulose and polyvinyl alcohol (PVA) doped with CuO nanoparticles. The optical properties of carboxymethyl cellulose (CMC), PVA/carboxymethyl cellulose and CuO/PVA/carboxymethyl cellulose films were found to be different, depending on their composition. It was observed than the optical transmittance (T%) of carboxymethyl cellulose increased to approximately 87% by adding PVA, but decreased to approximately 77% after doping with 0.5 wt % CuO NPs. The refractive index of CMC has reached 1.576 by adding PVA and 1.852 by doping with CuO NPs [40].

Kayani et al. reported an analysis of the transmission spectra of CuO NPs: at 400 °C the average size of the nanoparticles was 350 nm and at 1000 °C, 367 nm, corresponding to 3.38 eV and 3.54 eV, respectively [11].

El-Trass et al. also synthesized CuO NPs by using the alcohol-thermal method and it was observed by UV-Vis that the absorption bandwidth of bulk CuO (1.85 eV) is narrower than the bandwidth of the CuO NPs (2.36 eV) [41].

### 3.2. Magnetic Properties

The magnetic properties of CuO NPs are also influenced by their dimensions [8]. Moreover, the magnetic properties of CuO NPs strictly depend on their morphology [42]. In a study focused on the properties of nanoparticles, the authors have obtained CuO NPs with dimensions from 13 nm to 33 nm and they confirmed a weak ferromagnetic interaction, the process being slightly influenced by the size of the particles [8]. On the contrary, Bisht et al. reported that in the case of CuO NPs with dimensions between 9 and 16 nm, the peak present in zero field cooled magnetization is absent. Also, there was a bifurcation between the zero field cooled and field cooled systems and it was observed that the system shows hysteresis at room temperature. The authors reported that the associated peak in magnetic viscosity and the relaxation of magnetization are similar to other nanoparticle systems [43]. These studies indicate that the magnetic properties of CuO NPs may be influenced by the particle size, but are definitely also controlled by other aspects, possibly their synthesis method, composition and ratio of bulk material and other physico-chemical properties.

### 3.3. Electrical Conductivity

It was reported in the literature that the synthesis temperature might control the electrical conductivity of CuO NPs. Zhang et al. reported that when temperature increases from 300 °C to 700 °C during the synthesis, the electrical conductivity of the CuO NPs increases from 10^−6^ (Ω cm)^−1^ to 10^−5^ (Ω cm)^−1^, due to the removal of H_2_O vapor from the air [8].

Azimi and Taheri performed an assessment in order to investigate the electrical conductivity of CuO NPs. They used an aqueous solution of CuO NPs at different concentrations (0.12 g/L, 0.14 g/L, 0.16 g/L and 0.18 g/L), various temperatures of nanofluids, different particle sizes (89 nm, 95 nm, 100 nm and 112 nm) and concentrations of nanofluids obtained at the following temperatures: 25 °C, 35 °C, 45 °C and 50 °C. The authors revealed that electrical conductivity grew with the increase of temperature and nanoparticle concentration. It was also noted that the electrical conductivity increased until the dimensions of the nanoparticles reached 95 nm in diameter. This study demonstrated that there is a correlation between the values of electrical conductivity and nanoparticle size [44].

## 4. Medical Applications

CuO nanoparticles may have different applications depending on the various properties they manifest, which are highly influenced by their size, surface properties, optical and magnetic traits, the synthesis method being an important parameter for controlling all these and thus, their biological properties. Some of these applications include doping materials in semiconductors, such as chemical sensors, antimicrobial agents, catalyst for different cross coupling reactions, anti-cancer formulations, coating materials etc. Future biomedical applications of CuO NPs are focused intensively on disease detection and could present potential applications in many other areas, for example, in the detection of viruses in the human body [45]. In a recent study, Li et al. developed a highly sensitive and selective method for the detection of H1N1 flu virus. The principle of this method is based on labeling of antibodies by using CuO NPs. This approach was designed a sandwich complex made of CuO NPs labeled polyclonal antibody, able to detect and bind antigens represented by the H1N1 virus [46]. The method is an enzymatic chromogenic approach, belonging to the so-called enzyme linked immunosorbent assay (ELISA) methods, and proved to be highly sensitive and faster, as compared with other related methods.

### 4.1. Antibacterial Activity

Although the specific mechanism of the antimicrobial effect related with the use of CuO nanoparticles is not known, several of their mechanisms of action on bacterial cells have been discussed. Even if not specific to CuO nanoparticles, but for most oxide nanoparticles, Zhang et al. reported that the generation of reactive oxygen species (ROS) within bacterial cells is enhanced when using CuO-water suspensions [8].

The antibacterial activity of CuO NPs seems to be different depending on the particularities of bacteria cells. For examples, their cellular walls seem to impact the antimicrobial effect of CuO NPs, Gram character being a key aspect. It was reported that 100% of *E. coli* cells, which are Gram negative, were killed when a concentrations of CuO NPs higher than 9.5% was used, while for the Gram positive species *Staphylococcus aureus* the killing ability was lower [47]. It was also reported that CuO nanoparticles inhibit the growth of *E. coli*, *Pseudomonas aeruginosa*, and *S. aureus* in a time dependent manner, the utilized dose being, of course, the most important factor [48].

Goyal et al. also reported that the antimicrobial properties depend on the surface properties and size of nanoparticles. It seems that small particles with a large surface area have better antibacterial activity, as compared with larger ones. CuO NPs showed a major antimicrobial activity also against *Bacillus subtilis* [49]. El-Nahhal et al. tested the antibacterial activity of CuO NP-coated cotton dressings and CuS nanoparticle-coated cotton dressings. Both were inoculated with *E. coli* and *S. aureus* in order to compare the antimicrobial effect of the two coating systems in a Gram negative and Gram positive model, respectively. The results showed that the sample with CuO NPs presented higher antibacterial activity than the sample coated with CuS nanoparticles which showed no reduction in the viability of tested bacteria [50]. Devi et al. studied the antimicrobial activity of bulk, as-prepared and annealed CuO NPs against *E. coli*, *Proteus mirabilis*, *Klebsiella* spp., and their effect was comparable with the antimicrobial activity of gentamycin on these strains [9,51].

### 4.2. Toxicity of CuO Nanoparticles

CuO nanoparticles reveal different toxic activities in vitro and in vivo, when were tested on mammalian cells and on various animal models [52,53]. A study published in 1995 demonstrated that bioavailability of copper is the primary factor to determine the toxicity, a similar situation to what happens with toxic heavy metals [54]. Some features that can be modified to influence the toxicity of CuO NPs are:

(a) Size: small nanoparticles are more toxic than larger ones.
(b) Surface charge: the toxicity of nanoparticles is enhanced by a positive charge. This positive charge facilitates interactions between cells and nanoparticles.
(c) Dissolution: the dissolution of CuO NPs depends on the temperature and pH of the Solution and this has a major influence on their toxicity [55].

CuO NPs appear to be twenty times more toxic to the protozoan *Tetrahymena thermophila* as compared with their bulk material. It has been shown that the toxicity depends on the exposure time, the efficiency being maintained at maximum level between 4 and 24 h of exposure [4,56]. Also, in many studies the toxicity of nanoparticles of CuO was compared with their bulk form. Franklin et al. performed the first study on CuO NPs toxicity on algae. They used algal cultures exposed to various concentrations of the test substance. The experiment proved that CuO NPs were more toxic for algae than their bulk material. Their study revealed that toxic effects of CuO NPs were maintained for at least 72 h [54]. Another study, confirmed that the solubility and the toxicity of CuO NPs in artificial freshwater is higher than compared with their bulk form, their solubility being proposed to strictly influence the biological effect of these nanoparticles and thus, toxicity [57].

Saison et al. studied the toxicity of a core-shell formed by nanoparticles coated with polystyrene on *Chlamydomonas reinhardtii*. It was observed that this nanosystem was toxic for this algae and it was concluded that toxicity was caused not only by the type of nanoparticle, but also by the surface properties of these nanoparticles [58].

Another study was performed to determine the effects of CuO NPs on *Xenopus laevis* tadpoles through metamorphosis. CuO NPs with size ranging to 23–37 nm and surface area of 24–40 m^2^·g^−1^ were tested. The results revealed that the exposure to this nanoparticles for 5 days induced at least 40% mortality in all test groups [6]. Also, a high toxicity of these nanoparticles on *Cyprinus carpio* has been reported [59].

Even though the molecular toxicity mechanisms of CuO NPs on eukaryote models are not fully understood, many studies have shown that these nanoparticles promote mitochondrial damage, DNA damage and oxidative DNA damage [8].

In vitro assays revealed that toxicity of CuO NPs depended on the size and shape of the particles. For example, it was reported in literature that treatment with CuO NPs caused a significant decrease in cell proliferation, resulting in little cellular coverage of the culturing substrate after up to 6 days after the treatment. Moreover, the toxicity of CuO NPs is different depending on the differentiation state of the cells. CuO NPs toxicity seems to be significantly increased in differentiated cells as compared to non-differentiated cells.

In a recent study, Thit et al. aimed to determine the role of ROS release and establish the sequence of events during CuO NP toxicity. Their research utilized *N*-acetylcysteine to examine if increasing the cellular oxidative defense can mitigate DNA cytotoxicity and damage. The cells treated with Cu^2+^ did not show a significant increase in the death of the cells 48 h after experiment initiation.

Results showed that CuO NPs were more toxic than Cu^2+^, due to the increase in the generation of ROS, DNA damage and decrease levels of reduced glutathione (GSH) compared to control [60,61].

The exposure of different vertebrate embryos to CuO NPs containing 10 mg·Cu/L did not produce a significant decrease of embryo survival. However, the ionic form seemed to be the most toxic, altering all the analyzed parameters, including survival and malformation, especially when embryos were exposed to 5–10 mg·Cu/L [62].

CuO NPs showed relatively high toxicity for human lung cultured cells and for human skin organ cultures, when compared with their micron-sized alternatives [63,64]. Rafiei et al. reported that CuO NPs induced oxidative stress in various cultured cells [65]. Also, Karlsson et al. reported that the oxidative stress induced by CuO ions could also support its genotoxicity [66]. In another study, Sun et al. compared the cytotoxicity of CuO NPs, ZnO nanoparticles, Fe_2_O_3_ nanoparticles, Fe_2_O_4_ nanoparticles and Al_2_O_3_ nanoparticles in human cardiac microvascular endothelial cells (HCMECs). Cells were exposed to concentrations from 0.001 to 100 μg/mL of these nanoparticles for 12 to 24 h and the proliferation rates of these cells were analyzed by the reduction of tetrazolium salt (MTT) assay. Results showed that CuO and ZnO nanoparticles had a high degree of cytotoxicity against endothelial cells at all time points [67]. Dai et al. also investigated if the toxicity and bioaccumulation of sediment-associated Cu depends on the form of nanoparticles (CuO NPs vs. aqueous Cu, micron-sized particles) and nanoparticles shape (sphere-, rod-, or platelet-shaped CuO NPs) in a *Capitella teleta* worm model.

The results revealed no significant differences between measured sediment Cu concentrations; mortality rates were lower than 10% in worms grown in the presence of CuO NPs for up to 7 days. The results also revealed that worms were growing faster during the uptake period and slower during the depuration period, in all growth conditions and applied treatments [68].

Karlsson et al. studied the damage of cell membranes after treatment with CuO NPs and Cu metal nanoparticles. The authors observed that CuO NPs had caused very limited damage to the cell membrane, in comparison with Cu metal nanoparticles, which caused significant damage [63]. Also, Isani et al. reported that cell membrane damage caused by CuO NPs also increased hemolysis [15].

Fahmy and Cormier performed a study to analyze whether CuO NPs induce oxidative stress and cytotoxicity in airway epithelial cells. In this study, they were trying to compare the in vitro responses of respiratory epithelial cells after exposure to commercially available CuO NPs. They examined the ability of CuO NPs to generate ROS in HEP-2 cells, and compared the results with the ability of CuO NPs to induce oxidative stress in human epithelial cells. The authors observed that CuO NPs generated cytotoxicity, even at low doses, and were capable of inducing cell death [69]. In another study, Ahir et al. investigated the cytotoxicity of CuO NPs in HEP G2 cells and their results showed that these particles inhibit the growth of melanoma cells. CuO NPs fabricated with folic acid proved to be an efficient therapeutic option against triple negative breast cancer cells [70].

Chibber et al. assessed the neuronal toxicity of CuO NPs. After 24 h of exposure, some of the treated neurons partially detached from the dish and were found in suspension in the culture media. Detachment of the cells indicates the loss of the membrane integrity of neurons [71].

**Figure 2:**
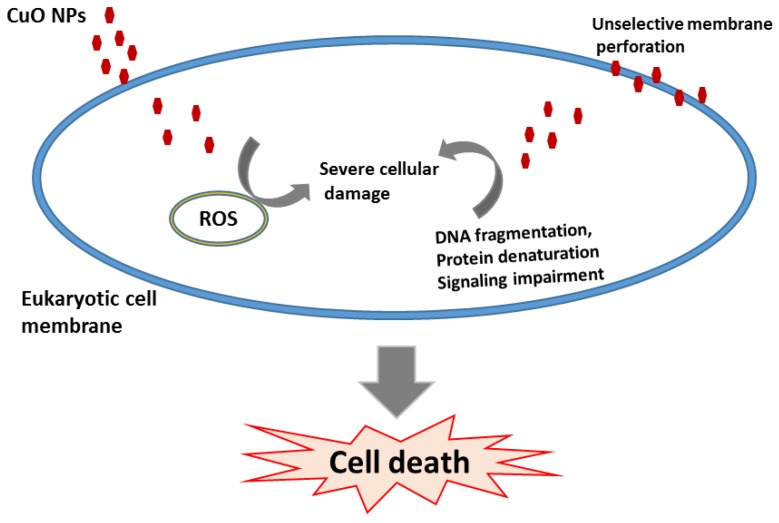
Toxicity mechanism of CuO nanoparticles in eukaryotic cells.

The mechanism of toxicity of CuO NPs in the cellular membrane was suggested to follow a Trojan horse-type mechanism (Figure 2). If these nanoparticles are soluble they can penetrate the membrane cancelling the barrier function of the membrane. After entering the cells, nanoparticles are able to dissolve at the acidic intracellular pH (4.5), and metal ions produce pores in the membrane [72,73].

### 4.3. Current Applications of CuO NPs

CuO nanoparticles are mainly utilized as antimicrobial agents. They are used in hospitals due to their antimicrobial ability to kill more than 99.9% of Gram-positive and negative bacteria within 2 h of exposure, if a suitable dose is applied. Studies reported that the utilization of CuO reduces the occurrence of hospital-acquired infections and the costs associated with health care in health care facilities. Bed sheets containing CuO NPs are considered one of the most interesting innovations in medical care, since they reduce microbial attachment and thus microbial infections within hospitals [74].

Previous work had demonstrated that CuO NPs also have beneficial effects on the skin. Studies conducted on women who utilized pillowcases and beddings containing CuO NPs revealed an improved aspect of the facial skin and an increase in the foot skin elasticity using socks impregnated with copper oxide nanoparticles [75]. Some advantages of using copper-oxide in hospital textiles are:

(1) It is effective against both, susceptible and antibiotic resistant microorganisms involved in nosocomial infections;
(2) It has wide antifungal spectrum and antibacterial properties;
(3) It inhibits biofilm or the development of microorganisms in attached communities on the surface of materials coated with CuO NPs;
(4) It does not cause skin irritation or sensitization;
(5) It is safe for humans if used externally and in low amounts [76].

Another application of these nanoparticles relies in their wound healing activity. Various wound dressings and textiles have been developed to treat burns and other skin injuries. The healing activity is proved to be strictly correlated with the capacity of CuO NPs to limit microbial colonization of the treated areas as well to avoid infection, while promoting regeneration of damaged tissue [77].

## 5. Conclusions and Perspectives

CuO nanoparticles can be synthesized by various methods, and by using various bulk materials and coating agents to obtain different types of nanosystems with various applications. All these aspects may significantly impact on their physico-chemical and biological properties and may affect their biomedical applications. The use of CuO nanoparticles in drug delivery formulations is still limited due to the enhanced toxicity, however other applications, such as topic formulations, dressings and coated textiles are of a great interest among the medical environments and others (i.e., cosmetics, textile industry etc.). The main application of such nanoformulations relies on their antimicrobial ability that allows for the development of multiple products, from antimicrobial solutions utilized to disinfect the surfaces and medical devices to antimicrobial wound dressings, textiles and coatings. For improving their applications on the biomedical field, researchers are striving to find optimal synthesis approaches in order to decrease the toxicity of CuO NPs but at the same time to keep or even improve their efficiency in diagnosis, therapy and, maybe even prophylaxis.

## Figures and Tables

**Table 1:** The synthesis of CuO NPs with different methods results in different sizes [10].

Preparation Method	Size (nm)
Electrochemical method	4
Sonochemical synthesis	20–30
Sol-gel techniques	7–9
Microemulsion system	5–25
Precipitation synthesis	4
Microwave irradiation	3–5
